# A New Non-invasive Neuromodulation Technique for Super Refractory Status Epilepticus: Can We Consider tDCS for This Devastating Condition?

**DOI:** 10.1007/s13311-022-01329-1

**Published:** 2022-12-09

**Authors:** Daniel San-Juan, Ioannis Stavropoulos, Antonio Valentin

**Affiliations:** 1 Epilepsy Clinic, National Institute of Neurology and Neurosurgery, Mexico City, Mexico; 2 Institute of Psychiatry, Psychology and Neuroscience, King’s College London, London, UK; 3 Department Of Clinical Neurophysiology, King’s College Hospital, London, UK

**Keywords:** Super-refractory status epilepticus, tDCS, Neuromodulation

Status epilepticus (SE) is a medical emergency that, despite medical treatment, can evolve to refractory (also called super-refractory [≥ 24 h]) status epilepticus (srSE) in 29–43% of cases [1]. This condition is associated with significant brain damage and mortality in a large percentage of patients. The srSE treatment, apart from standard 1st and 2nd line antiseizure drugs, can also include anesthetic agents, immunotherapy, plasmapheresis, hypothermia, ketogenic diet, and neuromodulation techniques either invasive (vagal nerve stimulation or deep brain stimulation) and/or non-invasive (transcranial magnetic stimulation or electroconvulsive therapy), but the published evidence remains insufficient [2–4]. The main advantage of trying neuromodulation techniques in patients with srSE is that these techniques could be used as adjuvants to standard pharmacological treatments without interfering with the potential drug benefits and may broaden the clinical decision-making time [5].

Transcranial direct electrical current stimulation (tDCS) is an emerging non-invasive neuromodulation technique, which applies a weak direct current stimulation through the scalp to induce linear and non-linear polarized effects at the subthreshold level in the neuronal membrane. Especially, cathodal stimulation induces hyperpolarization in neuronal bodies provoking acute and long-term effects that could be potentially relevant in the physiopathology of srSE. For example, tDCS could be potentially associated with an acute reduction in the excitatory presynaptic input or depression of synaptic force mediated by N-methyl-D-aspartate (NMDA) receptors, eliciting long-lasting effects including transmembrane protein migration and/or anti-inflammatory effects [6]. tDCS has been safely tested interictally in patients with different focal-onset refractory epilepsy types with good (50–70%) results in the reduction of interictal epileptiform discharges and seizure frequency. In particular, the acute effect of this neuromodulation technique has been noted in a small case series of three children with refractory focal epilepsy who underwent cathodal tDCS (2 mA, 30 min) with simultaneous EEG, showing a mean reduction of 58% of seizing events aborting the tonic seizures without any complications [7].

In this issue of *Neurotherapeutics*, Ng et al. [8] present the results of using high definition cathodal tDCS in ten adults with srSE secondary to several etiologies and diverse sedative and non-sedative pharmacological treatments. During this study, a 20-min session (2 mA) alongside a simultaneous EEG recording with different number of sessions (1–10) was used to evaluate the acute effects. Similar tDCS stimulation parameters have been previously described [9]. Against the baseline measurements, a reduction of 50% of median ictal epileptiform discharges rate, per patient and per session, was noted during the cathodal tDCS, and a reduction of 25% was noted in the period immediately following the intervention. Cathodal tDCS was safe and 90% of the patients were discharged from intensive care unit (ICU). Unfortunately, 70% of the patients in this study died during their hospitalization, and a similar number has already been previously published [4, 5].

Although the presenting findings are encouraging and this non-invasive technique looks promising for clinical use in this devastating condition, some issues are still noted for the use of neuromodulation in refractory epilepsy to be explored in future clinical trials. Firstly, the final morbidity and mortality outcomes in the study were not better than the ones described in the literature. As it is observed with other neuromodulation techniques [4], tDCS appears to facilitate the cessation of ictal activity allowing for discharge from ICU. Knowing the clinical implications from remaining in status epilepticus for long periods, someone arguably would wonder what the optimal time after srSE onset is to attempt a non-invasive neuromodulation technique, and whether this time period could alter overall outcome and not only the rate of discharge from ICU. Moreover, several confounding factors could possibly explain why some patients were not responders or worsened during the study, and several variables should be considered during studies of tDCS in srSE: (a) patients’ characteristics (focal or generalized seizures, previous epilepsy or new onset srSE, genetic variability, age, sex, special populations such as pregnancy); (b) pathological conditions such as reversible or irreversible etiologies and influence of pharmacological interventions; (c) tDCS montages (cephalic or non-cephalic, number of electrodes, stimulated lesion vs non-lesion regions); and (d) optimal parameters of stimulation (duration of a session, frequency of session, current intensity, optimal timing to apply, total number of sessions, stimulation focus choice).

Exploring treatment options for srSE has been proven difficult for many years. The well planned and performed study of Ng et al. [8] can set the basis for further multicenter clinical trials to assess the efficacy of tDCS or other neuromodulation techniques as potential add-on interventions in this life-threatening neurological condition, aiming to reduce ICU length of stay and total health outcomes.

## Supplementary Information

Below is the link to the electronic supplementary material.

Supplementary file1 (PDF 462 kb)

Supplementary file2 (PDF 606 kb)

Supplementary file3 (PDF 463 kb)

## Data Availability

Data sharing is not applicable to this commentary as no new data were created or analyzed in this study.
